# Phenotype, allele and genotype frequency distribution of ABO and Rh(D) blood group among blood donors attending regional blood transfusion centre in Delhi, India

**DOI:** 10.6026/97320630019385

**Published:** 2023-04-30

**Authors:** Sanjay Kumar Thakur, Sompal Singh, Dinesh Kumar Negi, Anil Kumar Sinha

**Affiliations:** 1 P.G. Department of Zoology, Veer Kunwar Singh University, Ara, Bihar 802301, India; 2 Department of Regional Blood Transfusion Centre and Pathology, Hindu Rao Hospital and NDMC Medical College and hospital, Delhi 110007, India

**Keywords:** Blood group, Phenotype, Genotype, Allele, Hardy-Weinberg equilibrium assumptions

## Abstract

The ABO and Rh blood group phenotypes, alleles, and genotype frequencies have many biological and medical implications. The frequency differs broadly according to races, geographical borders and ethnicity, even within the same region. This study was designed to determine the frequency of ABO and Rh blood groups among blood donors attending the regional blood transfusion centre in Delhi. The gel card method was used to determine the ABO and Rh(D) blood groups of donors who donated blood between January 1, 2020, and June 30, 2022. The assumption of Hardy-Weinberg equilibrium was used to determine allele and genotype frequencies of blood donors. A total of 16,925 blood units were donated during the study period. Donors phenotype frequencies of ABO were as follows: 'A' (23.88%), 'B' (37.38%), 'AB' (9.97%) and 'O '(29.27%). Rh(D)+Ve (D) were (94.9%) and Rh(D)-Ve (d) were (5.01%), which follow an order of B > O > A > AB and Rh-D > d for Rh. Donors ABO and Rh (D) allele frequencies were I^A^-0.183, I^B^-0.277, I^O^-0.541 and I^D^-0.776, I^d^-0.224 respectively. Allele frequencies follow an order of I^O^ > I^B^ > I^A^ and Rh- I^D^ > I^d^. Donors ABO genotype frequencies were AA-0.0333, AO-0.198, BB-0.0768, BO-0.30, AB-0.101, OO-0.293 and Rh(D) genotype frequencies were DD-0.602, Dd-0.347, dd-0.0501. Genotype frequencies follow an order of BO > OO > AO > AB > BB > AA and DD > Dd > dd. Among our donors, which were mostly from northern India, the ABO and Rh(D) blood groups have the highest proportion of ABO-B and Rh(D)+Ve and the lowest proportion of ABO-AB and Rh(D)-Ve, with a stable order of B > O > A > AB and D > d for phenotype, I^O^ > I^B^ > I^A^ and I^D^ > I^d^ for allele and BO > OO > AO > AB > BB > AA and DD > Dd > dd for genotype.

## Backgrounds:

The ABO blood group system was discovered by Karl Landsteiner in the year 1901 [1], which opened the door for blood transfusion and a wide range of discoveries in immunohematology. He was awarded the Nobel Prize for this work in 1930. In the year 1902, Alfred Von Decastello and Adriano Sturli discovered the fourth type of blood group AB [2]. The Rh (Rhesus factor) system was later described by both Landsteiner and Weiner in their joint work in 1940 [3]. Blood group antigens present on the plasma membrane of RBCs (red blood cells) are inherited. ABO and Rhesus Rh blood group determining antigens are carbohydrates and proteins bound to lipids or proteins in the plasma membrane of red cells. The ABO gene is located on the long arm of the ninth human chromosome (9q34.1) [4], while the Rh (D) gene, encoding the Rh protein, is located on chromosome 1p34-p36 [5]. Nearly 400 red blood cell antigens were described, and these are organized into 30 blood group systems by the International Society of Blood Transfusion (ISBT). In these blood group systems, ABO and Rhesus (Rh) are the most important clinically [6]. Blood transports oxygen, physiological waste products, nutrients and hormones in the body. In cases of deficiency of blood in the body, blood transfusion is required for survival. Blood group antigens play a vital role in compatible blood transfusion and safety. Blood group antigens A and B are highly antigenic and persons missing the corresponding antigen have naturally occurring antibodies in their plasma that can cause hemolysis in vivo after an incompatible blood transfusion. The second most antigenic and clinically significant blood group system is Rh, which has two phenotypes; Rh(D) positive (+Ve) and Rh (D) negative (-Ve), depending on the presence or absence of the D antigen on the red cell [3, 7]. The detection of the Rh antigen in pregnant women is important to avoid the erythroblastosis fetalis that causes severe hemolytic disease of the newborn (HDN) [3, 7]. transfusion of Rh (D)+Ve blood in a Rh (D)-Ve person can leads to a severe blood transfusion reaction after subsequent transfusion of Rh(D)+Ve blood due to production of antibody anti-D in Rh (D)-Ve person. Many authors have reported the association of blood group antigens with various diseases. A recent study on COVID-19 infection showed, that people with blood group 'A' are at higher risk, while those with blood group 'O' have a lower risk of death due to COVID-19 infection [8]. Study shows infectious diseases have associations with ABO blood group antigen, gastric ulcers have associations with blood group 'O', meningitis, peptic ulcers, oral candidiasis, and urinary tract infection with non-secretors blood group, malaria with A,B, and AB, leishmaniasis with ABO, typhoid and filariasis with 'B', smallpox with 'A', plague with 'O', Enterotoxoid mediated cholera with A and B and Glue ear with blood group 'A' [9]. Diseases such as coeliac disease, ankylosing spondylitis, Graves disease, and non-insulin dependent diabetes are associated with A,B non-secretors and gastro-duodenal ulcers with O non-secretor blood group antigen. Diseases such as capsular glaucoma and heart disease have an association with the A blood group, and ruptured Achilles tendon and parathyroid clear cell hyperplasia have an association with the O blood group [9]. Different types of carcinoma have tissue-specific changes in blood group antigen expression [9] and many types of cancer, such as rectal, cervical, pancreatic, leukaemia (ALL), gastric, breast, and ovarian cancers, have associations with 'A' blood group [9]. The ABO and Rh blood group phenotypes, alleles, and genotype frequencies differ broadly according to races, geographical borders, and ethnicity, even within the same region. The knowledge of ABO and Rh blood group frequency distribution in a particular population is very important for blood transfusion services and for physicians care of their patients. It helps to understand the deficiency of a particular group in a particular area, which helps to decide the way of mobilization of voluntary blood donors and encourage deficient group donors to donate more frequently. This is useful for health planners while making preventive measures in the particular region to face future health challenges. This is also important and useful for biological researchers for the study of inheritance patterns, population genetics, population migration patterns, disputed paternity cases, medicolegal issues, disease susceptibility, reliable geographical information, and anthropological and forensic studies in the population [3-9]. Therefore, it is of interest to determine the frequency and distribution of ABO and Rh(D) blood group patterns among blood donors in the Regional Blood Transfusion Centre, North Delhi.

## Methods and Materials:

### Ethical Considerations:

The study was started after the approval of the institutional ethical review committees of Hindu Rao Hospital and NDMC Medical College, Delhi, by approval number-F.No:IEC/NDMC/2021/69. The consent for blood donation was obtained from all the blood donors. For this study, only data from routine blood grouping of blood donors entered in blood bank inventory were used. There was no separate blood sample obtained from the donors for this study; hence, a separate informed consent for data analysis was not obtained from donors.

### Study Area and Design:

This study was carried out at the Regional Blood Transfusion Centre, situated in Delhi, India. The blood groups of voluntary and replacement blood donors during the period of 1st January 2020 to 31 June 2022 were studied. All the blood donors who qualify for blood donation as per the standard operating procedure of the blood bank, i.e healthy donors between the age group of 18 years to 65 years, and hemoglobin level >12.5 g/dL, were accepted.

### Sampling Technique and Laboratory investigations:

All the consecutive blood donors who donated blood during the study period were included in this study. A total of 17025 blood donors were included in this study. The blood samples of all the blood donors, collected in EDTA and plain tubes, were tested for ABO and Rh(D) blood groups. The ABO and Rh(D) blood groups were determined by the hem-agglutination method, using commercial Gel cards (DiaClon ABO/D+Reverse Grouping, BIO-RAD, Switzerland). The blood grouping test was performed according to manufacturer instructions. For the determination of A, B, O, D and d alleles, the Hardy Weinberg equilibrium assumption was used and expressed as a proportion.(see PDF)

### Data Collection:

According to the assumptions of Hardy-Weinberg equilibrium, in a large population from generation to generation, the allelic and genotypic frequencies will remain stable if the population has random mating, no mutation, no migration, and no natural selection. Although, in nature, Hardy-Weinberg equilibrium never occurs because there is always at least one condition for this rule to be violated, it provides an ideal baseline against which gene evolution in a population can be measured. The equations can be used on any population to measure their allelic and genotypic frequency. The equations can be used on any population to measure their allelic and genotypic frequency.

### Calculation of ABO gene frequency:

The ABO blood group gene locus is controlled by two co-dominants - I^A^, I^B^ and one recessive - I^O^ alleles.

Equations used to determine ABO allele frequency according to Hardy Weinberg equilibrium:

1. A (I^A^) + B(I^B^) + O(I^O^) = 1

Where A (I^A^) is the allele frequency of dominant gene A, B (I^B^) is the allele frequency of dominant gene B and O (I^O^) is the allele frequency of recessive O gene.

Frequency of AB phenotype = 2AB (I^A^ I^B^)

### Calculation Rh (D) gene frequency:

The Rh(D) blood group gene locus is controlled by one dominant- I^D^ and one recessive I^d^ allele. Equation applied to determine D and d allele frequencies according to Hardy Weinberg equilibrium:

3. D (I^D^) + d (I^d^) = 1 (see PDF)

4. D² (I^D^ I^D^) + 2Dd (I^D^ I^d^) + d² (I^d^ I^d^) = 1

Where D²(I^D^ I^D^) is frequency of dominant homozygous genotype, 2Dd (I^D^ I^d^) is of heterozygous genotype and d2 (I^d^ I^d^) is of homozygous recessive d genotype.

Phenotype frequency was calculated by using equation:

Frequency of Rh(D) positive phenotype = D² (I^D^ I^D^) + 2Dd (I^D^ I^d^)

Frequency of Rh(d) negative phenotype = d² (I^d^ I^d^)

### Statistical analysis:

Study data of donor were obtained from the blood bank inventory registers. The data was collected and entered into Microsoft Excel sheet and statistical analysis was performed using open source statistical software R. Analysis performed for descriptive statistics and frequency distribution. Results were presented as pie chart and tables. The observed allele and genotype frequencies of blood donors ABO and Rh(D) blood groups obtained by using Hardy-Weinberg equilibrium assumption and compared by using chi-square test. The *p-values* of less than 0.05 were considered statistically significant.

## Results:

**Figure 1:**
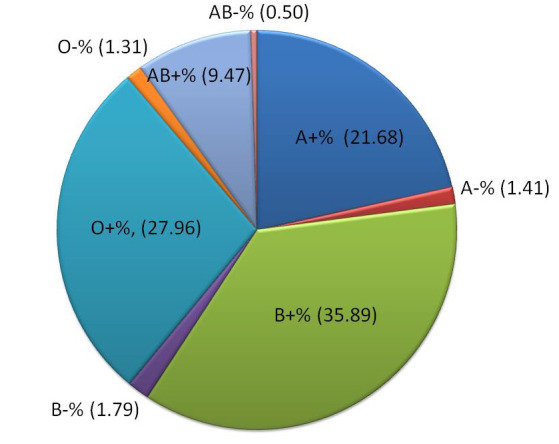
Percentage distribution of the ABO and Rh blood groups among blood donors.

Of a total of 16,925 healthy blood donors, the ABO and R(D) blood groups were determined. The number and percentage (Figure 1) of A+ve blood donors were 3669 (21.68%), A-ve were 238 (1.41%), B+ve were 6074 (35.89%), B-ve were 303 (1.79%), AB+ve were 1602 (9.47%), AB-ve were 85 (0.50%), O+ve were 4732 (27.96%) and O-ve were 222 (1.31%).

**Figure 2:**
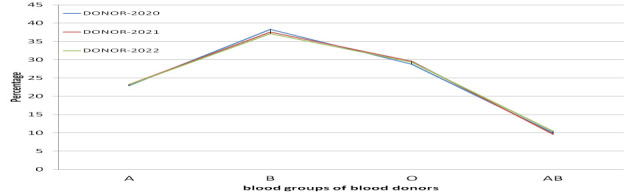
Year wise trend in frequency distribution of ABO blood group.

The number and percentage of 'A' blood group donors were 3907 (23.08%), 'B' were 6377 (37.68%), AB were 1687 (9.97%), 'O' were 4954 (29.27%), Rh(D) positive were 16077 (94.99%) and Rh(D) negative were 848 (5.01%). The percentages of ABO frequency follow an order of B > O > A > AB and D > d for Rh. The percentages of ABO frequency follow a similar trend over the years 2020, 2021 and 2022 (Figure 2).

## Hardy Weinberg assumption:

## Phenotype:

As calculated using Hardy Weinberg, the expected frequencies of 'A' group donors were 0.231 (Table 1), 'B' was 0.377, 'AB' was 0.0997, 'O' was 0.293, Rh (D) positive was 0.95 and Rh (D) negative was 0.05. The ABO phenotype frequency follows an order of B > O > A > AB and D > d. There was no significant difference in the phonotype frequency of ABO and Rh (D) blood groups between years; 2020 and 2021, 2020 and 2022, 2021 and 2022 (Table 2).

## Allele:

The allele frequencies of these donors were I^A^-0.183, I^B^-0.277, I^O^-0.541 and Rh (D) positive (I^D^)-0.776, Rh (D) negative (I^d^)-0.224. The ABO allele frequencies follow an order of I^O^ > I^B^ > I^A^ and Rh I^D^ > I^d^ (Table 1). There was no significant difference in the allele frequency of ABO and Rh (D) blood groups (Table 2) between years: 2020 and 2021, 2020 and 2022, 2021 and 2022.

## Genotype:

The genotype frequencies of these donors were AA-0.0333, AO-0.198, BB-0.0768, BO-0.3, AB-0.101, OO-0.293, Rh(D) positive-DD-0.602, Dd-0.347 and Rh(D) negative-dd-0.0501 (Table 1). The ABO genotype frequencies follow an order of BO > OO > AO > AB > BB > AA and Rh DD > Dd > dd. There was no significant difference in the genotype frequency of the ABO and Rh (D) blood groups between years; 2020 and 2021, 2020 and 2022, 2021 and 2022 (tables 1 and 2Table 1,Table 2).

## Discussion:

Our study results show that the frequency (Figure 1) of blood group B+Ve is the highest (35.89%) and that of AB-Ve is the lowest (0.50%). The proportion was highest for group B (37.68%) and lowest for group AB (9.97%). The Rh (D) positive (94.99%) was 19 times higher than the Rh (D) negative (5.01%) (Figure 2), and followed an order of B > O > A > AB for ABO and D > d for Rh.

According to Hardy-Weinberg equilibrium assumptions, we found that allele frequency was highest for I^O^ (0.541) and lowest for I^A^ (0.183), and alleles followed an order of I^O^ ≥ I^B^ > I^A^ for ABO. For the Rh (D), allele was higher for I^D^ (0.776) and lower for I^d^ (0.224), following an order of I^D^ > I^d^ (Table 1). The genotype frequency was highest for BO (0.30) and lowest for AA (0.0333), and followed an order of BO > OO > AO > AB > BB > AA for ABO. For Rh (D), It was highest for DD (0.602) and lowest for dd (0.0501), which follow an order of DD > Dd > dd (Table 1). The ABO and Rh (D) phenotype, allele, and genotype frequencies were similar over the years 2020, 2021, and 2022, and there was no significant difference (Table 2).

The studies reported from different states of India (Table 3) show heterogeneity and variations in the ABO and Rh (D) blood group frequencies state-wise. The author Agrawal A et al. [7] reported in 2014, after studying five different regions (northern, eastern, southern, western, and central), that India had an overall ABO frequency order of phenotype O > B > A > AB and allele I^O^ > I^B^ > I^A^. They had also reported that the northern region of India (Dehradun) shows an ABO frequency order of B > O > A > AB and a Rh (D) frequency order of D > d, which is similar to our ABO, Rh (D) phenotype, and allele frequency [7]. Our results are also similar to other studies in the populations of north Indian states, viz., Delhi, Jharkhand, Madhya Pradesh [10], Haryana [11], and also in Sola, Ahmadabad [6]. The population of southern India, viz., Andhra Pradesh [10], Surypet, Telangana [12], Puducherry [13], Karnataka [14], and Assam [10], a state of north-east India, shows an order of O > B > A > B and Rh D > d, whereas Shrinagar, Uttarakhand[10], a state of north-west India, shows an order of B > A > O > AB and Rh D > d.

Worldwide blood group frequency, outside of India, varies and shows heterogeneity (Table 4) countrywide. The neighboring countries of India, viz., Pakistan [12] and Bangladesh [15], have similar ABO and Rh (D) frequency orders (B > O > A > AB and D > d) compared to our study (North India), whereas Lahore, Pakistan [16] and Vietnam [17] have a higher proportion of O (O > B > A > AB and D > d), similar to the order of the southern Indian population. Some other countries have a higher proportion of A: Nepal [18], Jordan [19], the USA (Hispanic, including Mexican, Puerto Rican, and Cuban) [17], Korea [17], Japan [17], and Switzerland [20] show an order of A > O > B > AB and D > d. Outside of India, most of the country, viz., Britain [12], Iran [10], Iraq [10], Gaza Strip, Palestine [21], Ethiopia [10], Gondar, Ethiopia [22], Uganda [10], Libya [10], Nairobi area in Kenya [23], Lagos, Nigeria (Africa) [24], Mogadishu-Somalia [25], Chongqing, China [26], the United States [17] and Australia [27] show variable percentage frequency of ABO and Rh(D) with higher frequency of the O group in the order of O > A > B > AB and D > d.

The author, Sahar S. Hanania et al., in 2007, analyzed molecular genotyping by the PCR method and compared it with the Hardy-Weinberg equilibrium assumption and found a similar result [19]. Our study result shows that the allele frequency O (I^O^) has a higher proportion in the ABO blood group due to the heterozygous phenotype of blood group A and B which have genotypes AO and BO, respectively. Worldwide allele frequency of blood group 'O' has a higher proportion (Table 3,Table 4) and follows an order of O > A > B or O > B > A. The allele of Rh (D) has a higher proportion compared to antithetical Rh (d), with an order of D > d worldwide, which is similar to our study.

## Conclusion:

Among our donors, which were mostly from northern India, the ABO and Rh (D) blood groups have the highest proportion of B+Ve and the lowest proportion of AB-Ve, with a stable order of B > O > A > AB and D > d for phenotype, I^O^ > I^B^ > I^A^ and I^D^ > I^d^ for allele, and BO > OO > AO > AB > BB > AA and DD > Dd > dd for genotype.

## Authors' contributions:

The study's design was created by Sanjay Kumar Thakur, Sompal Singh, Dinesh Kumar Negi, and Anil Kumar Sinha. Sanjay Kumar Thakur carried out the literature review, collecting data, performing data analysis, and manuscript preparation. Each author contributed to the production of the manuscript as well as the interpretation and analysis of the data. The preparation and critical assessment of the final draught of the manuscript was equally performed by all authors.

## Funding:

Present research did not receive any grant from any funding agencies in the public, commercial, not-for-profit sectors.

## Figures and Tables

**Table 1:** Phenotype, Allele, and Genotype Frequency of ABO and Rh (D) among Blood Donors at Regional Blood Transfusion Centre, North Delhi. The calculated Hardy Weinberg frequencies are in parenthesis.

**ABO**	**Year**	**A**		**B**		**AB**	**O**	**Total**	**Rh(D)+Ve**		**Rh(D)-Ve**	**Total**
Phenotype Frequency	2020	1171		1961		514(0.1)	1471(0.287)	5117	4828		289	5117
		-0.229		-0.383				-1	-0.944		-0.0565	-1
	2021	1791		2899		746 (0.0966)	2284 (0.2959)	7720 (1)	7335		385	7720
		-0.232		-0.3755					-0.9501		-0.0499	-1
	2022	945		1517		427 (0.1045)	1199 (0.2933)	4088	3914		174	4088
		-0.2312		-0.3711				-1	-0.9574		-0.0426	-1
	Total	3907		6377		1687 (0.0997)	4954 (0.293)	16925 (1)	16077		848 (0.0501)	16925
		-0.231		-0.377					-0.95			-1
Allele Frequency	2020	-0.182		-0.283		-	-0.536	-1.001	-0.762		-0.238	-1
	2021	-0.1826		-0.2754		-	-0.5439	-1.002	-0.7767		-0.2233	-1
	2022	-0.1826		-0.2735		-	-0.5416	-0.9977	-0.7937		-0.2063	-1
	Total	-0.183		-0.277		-	-0.541	-1.001	-0.776		-0.224	-1
Genotype		AA	AO	BB	BO	AB	OO		DD	Dd	dd	
Genotype Frequency	2020	-0.0333	-0.196	-0.08	-0.303	-0.103	-0.287	-1.0023	-0.581	-0.362	-0.057	-1
	2021	-0.0333	-0.1986	-0.0759	-0.2996	-0.1006	-0.2959	-1.004	-0.6032	-0.3469	-0.0499	-1
	2022	-0.0334	-0.1978	-0.0748	-0.2963	-0.0999	-0.2933	-0.9955	-0.6299	-0.3275	-0.0426	-1
	Total	-0.0333	-0.198	-0.0768	-0.3	-0.101	-0.293	-1.0021	-0.602	-0.347	-0.051	-1

**Table 2:** Result of Chi square test and p value for year wise difference in phenotype, allele and genotype frequency.

	**Year**	**ABO**		**Rh(D)**	
		χ²	p value	χ²	p value
Phenotype Frequency	2020 and 2021	0.00028	0.99999	0.00042	0.98347
	2020 and 2022	0.00036	0.99999	0.00048	0.9825
	2021 and 2022	0.00034	0.99999	0	0.99905
Allele Frequency	2020 and 2021	0.00016	0.99991	0.0006	0.98031
	2020 and 2022	0.00018	0.9999	0.0029	0.95699
	2021 and 2022	0	0.99999	0.00085	0.97664
Genotype Frequency	2020 and 2021	0.0003	0.99999	0.00114	0.99942
	2020 and 2022	0.00035	0.99999	0.00565	0.99717
	2021 and 2022	0	1	0.00171	0.99914

**Table 3:** Frequency of ABO and Rh(D) blood group in India and other countries.

**States of India**	**Year**	**Sample size**	**A%**	**B%**	**AB%**	**O%**	**Rh(D+)%**	**Rh(D-) %**	**Order of ABO frequency**
Delhi (present study)	2022	16925	23.08	37.68	9.97	29.27	94.99	5.01	B>O>A>AB
North India (Dehradun) [7]	2014	2042	24.53	34.47	11.55	29.43	94.8	5.19	B>O>A>AB
Delhi[10]	2016	15446	22.6	37.8	10.1	29.5	94.47	5.53	B>O>A>AB
Jharkhand [10]	2016	2055	22.09	35.15	8.03	34.73	96.46	3.54	B>O>A>AB
Madhya Pradesh [10]	2016	800	25.63	39.25	6.5	28.63	94.88	5.12	B>O>A>AB
Haryana [11]	2016	3202	22.21	37.82	9.15	30.82	91.07	8.93	B>O>A>AB
Sola, Ahmadabad [6]	2012	53160	21.94	39.4	7.86	30.7	95.05	4.95	B>O>A>AB
Andhra Pradesh [10]	2016	6942	20	35.8	7.3	36.9	96.28	3.72	O>B>A>AB
Surypet, Telangana [12]	2019	7035	17.95	32.5	5.62	43.9	95.72	4.27	O>B>A>AB
Puducherry [13]	2019	390	20	35	9	36	-	-	O>B>A>AB
Karnataka [14]	2014	43,103	25.8	27.3	4.8	42	94.64	5.35	O>B>A>AB
Assam[10]	2016	334	21.6	29.3	4.8	44.3	98.5	1.5	O>B>A>AB
Shrinagar, Uttarakhand[10]	2018	9883	30.39	31.68	11.7	26.24	93.51	6.49	B>A>O>AB
India(include 5 regiona) [7]	2014	10,000	22.88	32.26	7.74	37.12	94.61	5.39.	O>B>A>AB
South India (Chennai)[7]	2014	1808	20.68	33.07	6.25	38.99	93.91	6.08	O>B>A>AB
East India (Kolkata) [7]	2014	1595	21.88	33.85	6.7	37.55	95.23	4.76	O>B>A>AB
West India (Mumbai)[7]	2014	2220	23.69	32.74	6.8	36.75	92.97	7.02	O>B>A>AB
Central India (Nagpur)[7]	2014	2021	23.1	26.57	7.07	43.24	96.23	3.72	O>B>A>AB

**Table 4:** Frequency of ABO and Rh(D) blood group in other countries.

**Countries**	**Year**	**Sample size**	**A%**	**B%**	**AB%**	**O%**	**Rh(D+)%**	**Rh(D-)%**	**Order of ABO frequency**
Pakistan [12]	2008	22897	27.92	32.4	10.58	29	90.13	9.87	B>O>A>AB
Bangladesh[15]	2016	937	26.57	34.11	9.61	29.67	90.82	9.18	B>O>A>AB
Lahore, Pakistan [16]	2014	3000	24.2	37.8	9.1	28.8	93	7	O>B>A>AB
Vietnamese [17]	2004	9,024	22.5	27.9	5.8	43.8	99.1	0.9	O>B>A>AB
Nepal [18]	2000		34	29	4	33	96.7	3.3	A>O>B>AB
Jordan[19]	2007	12215	38.36	18.04	6.98	36.62,	-	-	A>O>B>AB
Hispanic (USA) [19]	2004	2,59,233	31.1	9.9	2.5	56.5	92.7	7.3	A>O>B>AB
Korean [19]	2004	15,817	32.2	27	10.7	30.1	99.5	0.5	A>O>B>AB
Japanese [19]	2004	16,154	37.7	20.4	8.5	33.4	98.1	1.9	A>O>B>AB
Switzerland[20]	2017	1,75,202	46.2	10.4	4.1	39.3	84.2	15.8	A>O>B>AB
Britain [12]	1993	-	42	8	3	47	83	17	O>A>B>AB
Iran[10]	2016	29922	28.48	24.71	6.6	40.21	92.38	7.62	O>A>B>AB
Iraq[10]	2016	1268	23.11	21.45	7.41	48.03	88.56	11.44	O>A>B>AB
Gaza strip, Palestine [21]	2007	14916	33.1	21.3	7.5	38.1	83.3	10.7	O>A>B>AB
Ethiopia[10]	2016	6922	31.9	21.5	3.5	43.1	92.8	9.877	O>A>B>AB
Gondar, Ethiopia [22]	2022	6471	26.44	21.71	4.81	47.04	94.24	5.76	O>A>B>AB
Uganda[10]	2016	23504	25	20.39	4.25	50.36	97.97	2.03	O>A>B>AB
Libya[10]	2016	1306	31.17	23.43	8.96	37.44	83.92	16.07	O>A>B>AB
Nairobi area in Kenya.[23]	1992	38,898	26.2	22	4.4	47.4	96.1	3.9	O>A>B>AB
Lagos, Nigeria (Africa) [24]	2016	11,911	21.37	17.57	2.37	58.7	95.6	3.4	O>A>B>AB
Mogadishu-Somalia[25]	2022	1811	27	10	2	61	97	3	O>A>B>AB
Chongqing, China [26]	2020	7,95,698	31.9	24.14	8.42	35.54	99.45	0.55	O>A>B>AB
